# Impella Induced Massive Hemolysis: Reemphasizing Echocardiographic Guidance for Correct Placement

**DOI:** 10.1155/2015/464135

**Published:** 2015-01-27

**Authors:** Shaun Cardozo, Tasneem Ahmed, Kevin Belgrave

**Affiliations:** Division of Cardiology, Department of Internal Medicine, Detroit Medical Center, Harper University Hospital, Wayne State University, Detroit, MI 48201, USA

## Abstract

The Impella LP 2.5 (Abiomed, Danvers, MA) has been a tool of use for high risk coronary procedures and for cardiogenic shock. As with any invasive or intracardiac device, improper placement can result in disastrous complications. Hemolytic anemia secondary to Impella implantation is one of the documented complications. However, cases of severe hemolytic anemia are rare in the literature. Proven imaging modalities like ultrasound need to be used to guide proper placement. We present a case of device induced severe hemolysis due to Impella insertion and the need to use ultrasound guidance to avoid such an unnecessary complication.

## 1. Introduction

Ventricular assist devices are becoming more common in the era of high risk coronary interventions. The use of the Impella is becoming more routine as opposed to the intra-aortic balloon pump for both patients undergoing high risk inventions and cardiogenic shock. Although the Impella can help improve circulatory support, the device itself can have complications. The potential risk of complications can be reduced with proper imaging, especially echocardiography, during implantation. Potential complications due to improper placement include hemolysis, mitral stenosis, and aortic regurgitation. We present a case of significant hemolysis secondary to improper positioning and resolution after correct echocardiographic guidance.

## 2. Case

Our patient is a 59-year male with history of ischemic cardiomyopathy (ejection fraction of 10%), end stage renal disease (ESRD), and prior internal cardiac defibrillator (ICD) placement who presented for an ICD generator change. During the procedure, the patient went into an episode of ventricular fibrillation and required resuscitative measures including external defibrillation and initiation of multiple pressors for blood pressure support. Due the severe LV dysfunction and hypotension, the decision was made for Impella insertion for hemodynamic circulatory support. After Impella insertion, placement was confirmed by fluoroscopy and the patient was transferred under guarded condition to the cardiac care unit.

**Figure 1:**
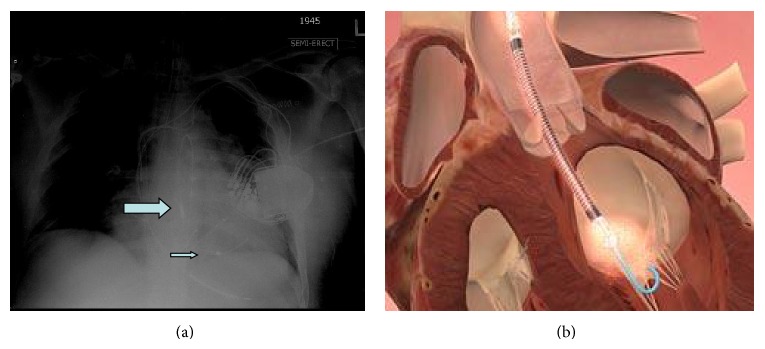
(a) An anteroposterior view of the chest showing the Impella inlet (marked with arrow) and outlet (marked with small arrow) below the aortic valve level. (b) Correct placement of Impella with supravalvular outlet (Courtesy of ABIOMED).

**Figure 2:**
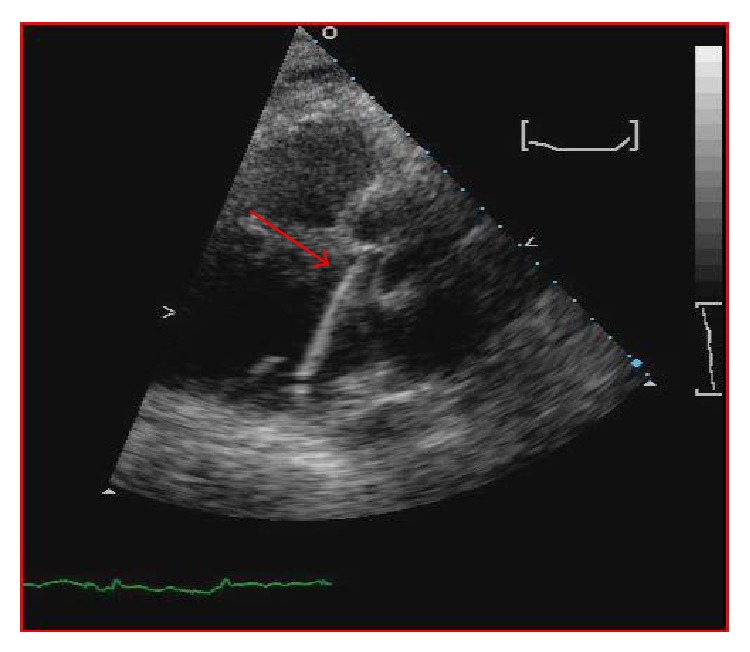
Parasternal long axis view showing the Impella catheter tip resting on the inferior basal wall (red arrow).

**Figure 3:**
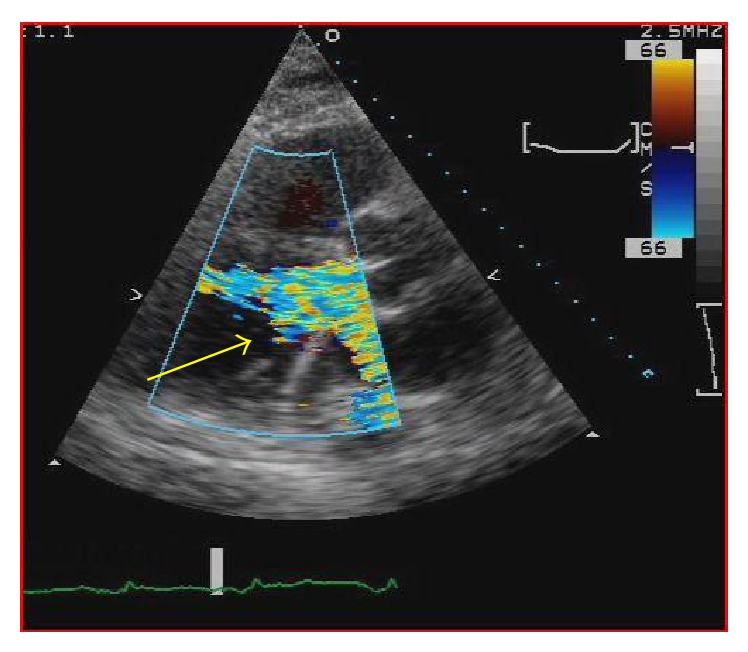
Parasternal long axis view with color flow showing the turbulence of the Impella located subvalvular (yellow arrow).

**Figure 4:**
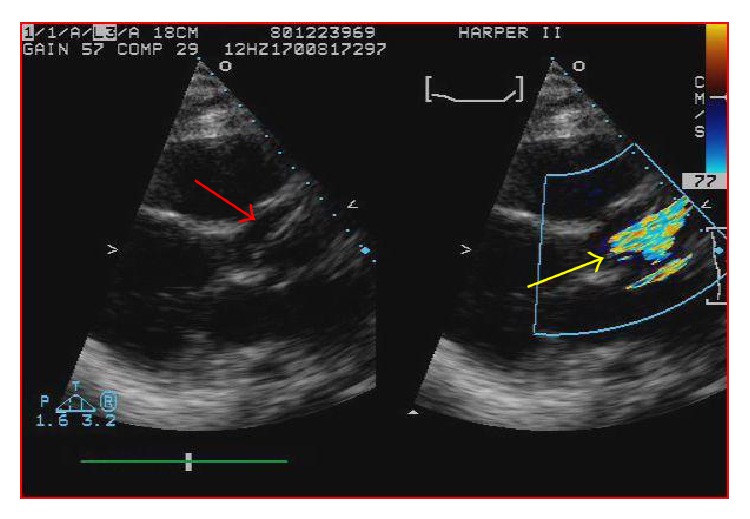
After echocardiographic guidance the Impella inlet is properly positioned above the aortic valve (red arrow). Supravalvular turbulence is indicated by the yellow arrow.

One day later the patient's hemoglobin fell to 8.4 g/dL and the potassium level increased to 6.5 mMol/L and subsequently nephrology was consulted. When the patient's lactate level returned at 2041 units/L, the concern for device induced hemolysis was proposed. A repeat chest X-ray indicated that the Impella catheter might be malpositioned within the left ventricular cavity (Figure 1). The cardiology team immediately performed a transthoracic echocardiogram (TTE) to confirm Impella placement. The TTE showed that the pigtail catheter tip was sitting on the basal inferior lateral wall and on color Doppler the inlet and outlet of the device were both subvalvular (Figures 2 and 3). The mispositioning of the Impella was inducing turbulence within the cavity and was the probable cause of the significant hemolysis. Under echocardiographic guidance, the Impella was withdrawn so that the outlet was supravalvular within the aortic root which was confirmed on color Doppler (Figure 4). After hemodialysis was performed the potassium level normalized and the hemoglobin stabilized. After careful evaluation on repeat laboratory testing, there was no evidence of significant hemolysis after repositioning of the device. At discharge, the final documented hemoglobin level was 11.9 g/dL and the lactate level was 512 units/L.

## 3. Discussion

The Impella is a commonly used left ventricular assist device for cases of cardiogenic shock and high risk revascularization procedures. Its success has especially been shown in the subgroup of unstable patients undergoing high risk left main stenting [1]. The literature does describe a few complications associated with the Impella device including sensor failure, pump displacement, and hemolysis [2]. While hemolysis is a documented complication, the incidence is rare, especially clinically significant hemolysis. There are two published cases of significant hemolysis secondary to Impella use [3, 4]. In both of those cases, the Impella was removed with improvement and resolution of the hemolysis. The hemolysis in those cases appeared to be secondary to the sheer stress caused by the device itself. In our patient, the hemolysis was a combination of the device and the improper positioning of the outlet in the subvalvular area. Proper repositioning with TTE guidance reduced the significant hemolysis and resolved the issue. Prior studies have supported echocardiographic guidance of the Impella assist devices because even correct placement of the inlet can still cause anatomic obstructions within the left ventricular cavity [5]. One published case describes the malpositioning of the Impella inducing functional mitral stenosis confirmed by TTE that was corrected after echocardiographic guidance for positioning [6]. Although malpositioning of the Impella device is rare, echocardiography remains a proven modality to confirm placement and avoid unnecessary complications. Our case highlights the need for echocardiographic guidance over fluoroscopy for optimal placement of the device and minimizing complication.

## References

[1] Sjauw K. D., Konorza T., Erbel R. (2009). Supported high-risk percutaneous coronary intervention with the Impella 2.5 device: the Europella registry. *Journal of the American College of Cardiology*.

[2] Meyns B., Dens J., Sergeant P., Herijgers P., Daenen W., Flameng W. (2003). Initial experiences with the Impella device in patients with cardiogenic shock-Impella support for cardiogenic shock. *Thoracic and Cardiovascular Surgeon*.

[3] Tanawuttiwat T., Chaparro S. V. (2013). An unexpected cause of massive hemolysis in percutaneous left ventricular assist device. *Cardiovascular Revascularization Medicine*.

[4] Sibbald M., Džavík V. (2012). Severe hemolysis associated with use of the impella LP 2.5 mechanical assist device. *Catheterization and Cardiovascular Interventions*.

[5] Catena E., Milazzo F., Merli M. (2004). Echocardiographic evaluation of patients receiving a new left ventricular assist device: the Impella recover 100. *European Journal of Echocardiography*.

[6] Toggweiler S., Jamshidi P., Erne P. (2008). Functional mitral stenosis: a rare complication of the Impella assist device. *European Journal of Echocardiography*.

